# College students’ anxiety after returning to school during the COVID-19 epidemic: What should we care

**DOI:** 10.1097/MD.0000000000032068

**Published:** 2022-12-02

**Authors:** Ting Ding, Chenjie Zhu, Linling Jing, Shanshan Gu

**Affiliations:** a Zhejiang Business College, Hangzhou, China; b Daishan first People’s Hospital, Zhejiang, China.

**Keywords:** anxiety, care, college, COVID-19 epidemic, health, student

## Abstract

The college students’ anxiety during the Coronavirus disease 2019 (COVID-19) epidemic remains unclear. We aimed to evaluate the college students’ anxiety after returning to school during the COVID-19 epidemic, to provide reference for the management and nursing care of college students. We conducted a survey from September 15, 2021 to September 30, 2021 investigate the anxiety level of college students. The Self-rating Anxiety Scale was used for anxiety assessment. The Spearman correlation analysis was conducted to evaluate the correlation between students’ anxiety and characteristics. Logistic regression analysis was used to explore the influencing factors of concurrent anxiety among college students. A total of 2168 college students were included, the incidence of anxiety was 30.07% in college students during the COVID-19 epidemic. Pearson correlation analyses showed that grade (*R* = 0.715), main use of computer and mobile phone (*R* = 0.622), daily exercise (*R* = 0.735), whether relatives or friends are infected with COVID-19 (*R* = 0.735) are associated with the anxiety level of college students (all *P* < .05). Logistic regression analysis indicated that senior year (Odds ratio [OR] = 2.064, 95% confidence interval [CI]: 1.355–3.001), online game (OR = 3.122, 95% CI: 2.562–3.899), relatives or friends are infected with COVID-19 (OR = 2.987, 95% CI: 1.901–3.451) are the independent risk factors of anxiety in college students (all *P* < .05). Daily exercise (OR = 0.514, 95% CI: 0.205–0.814) was the independent protective factors of anxiety in college students (*P* = .008). During the COVID-19 epidemic, college students have increased anxiety and there are many influencing factors. Administrators and educators should especially pay attention to the mental health of students with those risk factors to maintain students’ physical and mental health.

## 1. Introduction

The Coronavirus disease 2019 (COVID-19) epidemic continues to spread around the world due to its diverse transmission routes, strong infectivity, and high prevalence.^[1,2]^ The novel coronavirus is highly contagious and spreads rapidly, posing a serious threat to the health of the people.^[3]^ In the face of the increasingly severe epidemic, in order to curb the spread of the COVID-19 epidemic, the Chinese national government and various local departments have successively issued a number of policies to control population movement, and advocated the public to isolate at home.^[4–6]^ The isolation at home and the postponement of the start of school have brought an unprecedented “extended” vacation to college students.^[7,8]^ The impact of COVID-19 lockdown on physical, mental, and social wellbeing of elderly and fragile populations such as those of adolescents in specific countries such as Italy cannot be ignored.^[9,10]^ There is a certain sense of limitation, and the extended vacation can inevitably disrupt their study plans. Factors such as long-term home isolation, fear of the epidemic, and lack of knowledge about the COVID-19 have increased the psychological burden of college students, causing them to generate psychological stress, mainly manifested as panic, anxiety, depression and other emotions.^[11–13]^

Previous studies^[14,15]^ have shown that compared with their peers, college students have more worrisome health conditions and higher risks of psychiatric disorders, especially anxiety and depression. If the psychological stress of college students cannot be dealt with in a timely and correct manner, it will affect the physical and mental development of college students and even cause group harmful incidents.^[16]^ It’s been reported that anxiety and depression in college students may be frequently associated with suicidal behavior which is frequently underreported.^[17]^ Unfortunately, above 2% of the traffic accidents are suicide behaviors,^[18]^ and this phenomenon may be underreported considering that suicides by car accidents are reported as accidental in the national statistics. Understanding the anxiety of college students after returning to school and its influencing factors plays an important role in college students’ emotional management and mental health.^[19,20]^ Therefore, this study aimed to evaluate the anxiety status of college students after returning to school during the COVID-19 epidemic, and analyzed its influencing factors, so as to provide evidence for the psychological nursing and care for college students during the COVID-19 epidemic. We have made the main hypotheses that anxiety in college students after returning to school during the COVID-19 epidemic is increased and influenced by many factors, early targeted nursing care and interventions are needed.

## 2. Methods

### 2.1. Ethical consideration

In this study, all methods were performed in accordance with the relevant guidelines and regulations. The study protocol had been verified and approved by the mental health guidance center of Zhejiang Business College (approval number: 2021zsy-kj-03), and written informed consents had been obtained from all the participants.

### 2.2. Study population

From September 15, 2021 to September 30, 2021, we used the convenience sampling method to investigate the anxiety level of students in our college. The inclusion criteria of the survey respondents were as following: students were studying full-time in our college; the students were well informed and volunteered to participate in this survey. All respondents were informed of the purpose of the survey before the survey. The questionnaires were filled out by the students anonymously.

### 2.3. Survey content

*The Self-rating Anxiety Scale (SAS):* SAS was compiled by Zung in 1971 used for anxiety assessment.^[21]^ There are 20 questions in the scale, and each question uses a 4-point scoring method, corresponding to 1 to 4 points respectively. Among them, 1 to 4 are “no or very little time,” “a small part of the time,” “a considerable amount of time” and “most or all of the time.” The rough score of the scale multiplied by 1.25 is rounded to be the standard score. Previous studies have shown that SAS has good reliability and validity, with an internal consistency coefficient of 0.77 and a split-half reliability coefficient of 0.72.^[22,23]^ The standard score < 50 means no anxiety, 50 to 60 means mild anxiety, and 60 to 70 means moderate anxiety, ≥ 70 points means severe anxiety. The higher the score, the higher level of anxiety.

*Self-compiled questionnaires:* The researchers self-compiled questionnaire to collect the following information from the respondents: gender, age, grade, daily use time of computer and mobile phone (hours), main use of computer and mobile phone, daily exercise, sleep time per day (hours), whether relatives or friends are infected with COVID-19, whether pay attention to the epidemic every day.

After obtaining the informed consent of the research subjects, we introduced the purpose and significance of this investigation. We instructed them to answer truthfully based on their own situation in the past 2 weeks. All questions were set as mandatory questions, and each user can only participate once. The questionnaires adopted a unified guide language. The same IP address can only be answered once. The questionnaire did not involve private information such as names, and sensitive language was avoided. All the questionnaires could not be submitted before they were completed. The online questionnaire background automatically detected the answering time of each questionnaire, and removed the questionnaires whose answering time was less than 100 seconds.

### 2.4. Statistical analysis

After checking the collected data, we input the data to SPSS 22.0 software SPSS Statistics version 22.0 (IBM SPSS Inc., Chicago) for data analysis and processing. The enumerated data were expressed as the number of cases and percentage, and the measurement data are expressed as “mean ± standard deviation”. The Spearman correlation coefficient was used to evaluate the degree of correlation between students’ anxiety scores and characteristics. Logistic regression analysis was used to explore the influencing factors of concurrent anxiety among college students. The test level of this study was *α* = 0.05, and *P* < .05 indicated that the difference between the groups was statistically significant.

## 3. Results

### 3.1. The characteristics of included college students

**Figure 1.**
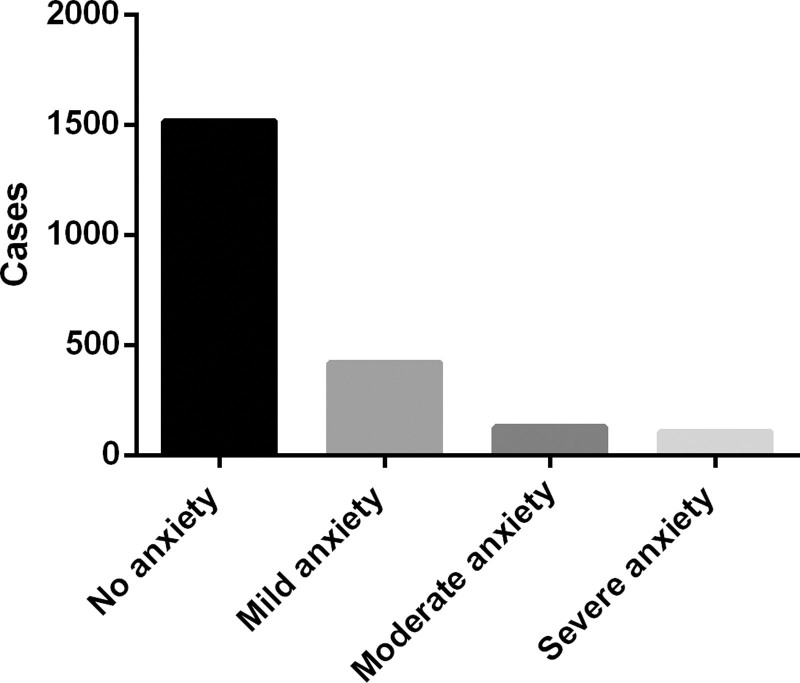
The anxiety level of college students during the COVID-19 epidemic.

A total of 2185 questionnaires were distributed in this study, 17 invalid questionnaires were excluded, and 2168 valid questionnaires were finally obtained. The average age of the respondents was (22.16 ± 2.59) years old, including 1022 (47.14%) males and 1146 (52.86%) females. As presented in Figure 1, the results of SAS assessment showed that 652 (30.07%) college students were anxious, among which 419 (19.33%) students had mild anxiety, 128 students (5.90%) had moderate anxiety, 105 (4.84%) students had severe anxiety.

As showed in Table 1, there were significant statistical differences on the anxiety level in students with different grade, daily use time of computer and mobile phone, main use of computer and mobile phone, daily exercise, whether relatives or friends are infected with COVID-19 (all *P* < .05). There were no significant statistical differences on the anxiety level in students with different gender, age, pay attention to the epidemic every day (all *P* > .05).

**Table 1:** The characteristics of included college students.

Variables	Cases	SAS	*F*/*t*	*P*
Gender			4.069	.093
Male	1022 (47.14%)	45.16 ± 15.42		
Female	1146 (52.86%)	46.22±16.39		
Age (y)			3.108	.056
<20	785 (36.21%)	44.94±14.25		
20–25	1206 (55.63%)	46.01±13.39		
>25	177 (8.16%)	46.47±17.04		
Grade			2.014	.012
Freshman	654 (30.17%)	43.09±15.33		
Sophomore	566 (26.11%)	44.12±16.28		
Junior year	512 (23.62%)	45.61±15.95		
Senior year	436 (20.10%)	48.02±20.37		
Daily use time of computer and mobile phone (h)			2.445	.046
<3	359 (16.56%)	44.11±16.85		
3–6	808 (37.27%)	45.03±18.25		
>6	1001 (46.17%)	46.96±21.42		
Main use of computer and mobile phone			3.182	.009
e-learning	873 (40.27%)	45.25±16.22		
Video	566 (26.11%)	45.09±15.26		
Online chatting	415 (19.14%)	44.12±16.01		
Online game	314 (14.48%)	48.19±19.75		
Daily exercise			2.866	.019
Yes	1145 (52.81%)	45.14±16.31		
No	1023 (47.19%)	46.56±15.29		
Sleep time per day (h)			4.079	.085
<6	480 (22.14%)	46.42±18.20		
6–8	611 (28.18%)	45.29±17.06		
8–10	536 (24.72%)	45.11±18.05		
>10	541 (24.95%)	45.98±17.53		
Whether relatives or friends are infected with COVID-19			1.803	.001
Yes	33 (1.52%)	48.69±10.31		
No	2135 (98.48%)	45.12±15.26		
Pay attention to the epidemic every day			1.188	.092
Yes	1554 (71.68%)	46.18±16.28		
No	614 (28.32%)	45.25±15.06		

SAS = self-rating anxiety scale.

### 3.2. Correlation analysis

As indicated in Table 2, Pearson correlation analyses showed that grade (*R* = 0.715), main use of computer and mobile phone (*R* = 0.622), daily exercise (*R* = 0.735), whether relatives or friends are infected with COVID-19 (*R* = 0.735) are associated with the anxiety level of college students (all *P* < .05).

**Table 2:** Pearson correlation analysis on the characteristics of students and anxiety.

Variables	*r*	*P*
Gender	0.114	.086
Age	0.076	.103
Grade	0.715	.011
Daily use time of computer and mobile phone (h)	0.294	.057
Main use of computer and mobile phone	0.622	.029
Daily exercise	0.735	.007
Sleep time per day (h)	0.207	.123
Whether relatives or friends are infected with COVID-19	0.811	.016
Pay attention to the epidemic every day	0.112	.109

COVID-19 = Coronavirus disease 2019.

### 3.3. Logistic regression analysis

The variable assignments of multivariate logistic regression on the anxiety are showed in Table 3. As indicated in Table 4, senior year (Odds ratio [OR] = 2. 064, 95% CI: 1.355–3.001), online game (OR = 3.122, 95% CI: 2.562–3.899), relatives or friends are infected with COVID-19 (OR = 2.987, 95% CI: 1.901–3.451) are the independent risk factors of anxiety in college students after returning to school during the COVID-19 epidemic (all *P* < .05). Daily exercise (OR = 0.514, 95% CI: 0.205–0.814) was the independent protective factors of anxiety in college students after returning to school during the COVID-19 epidemic (*P* = .008).

**Table 3:** The variable assignments of multivariate logistic regression on the anxiety.

Factors	Variables	Assignment
Anxiety	*Y*	Yes = 1, no = 2
Grade	*X*_1_	Senior year = 1, Junior year = 2, Sophomore = 3, Freshman = 4
Main use of computer and mobile phone	*X*_2_	Online game = 1, e-learning = 2, video = 3, online chatting = 4
Daily exercise	*X*_3_	Yes = 1, no = 2
Whether relatives or friends are infected with COVID-19	*X*_4_	Yes = 1, no = 2

COVID-19 = Coronavirus disease 2019.

**Table 4:** Logistic regression analysis on the influencing factors of anxiety in college students after returning to school during the COVID-19 epidemic.

Variables	β	SE	OR	95% CI	*P*
Senior year	0.103	0.211	2.064	1.355–3.001	.012
Online game	0.115	0.306	3.122	2.562–3.899	.026
Daily exercise	0.121	0.193	0.514	0.205–0.814	.008
Relatives or friends are infected with COVID-19	0.265	0.411	2.987	1.901–3.451	.033

95% CI = confidence interval, COVID-19 = Coronavirus disease 2019, OR = odds ratio.

## 4. Discussions

Understand the anxiety status of college students and their influencing factors during the COVID-19 epidemic can prevent the occurrence of anxiety, guide students to effectively regulate anxiety, and provide reference for the education department to issue relevant policies. This study shows that during the COVID-19 epidemic, the detection rate of anxiety among the surveyed college students in the past 2 weeks was 30.07%, which is slightly higher than previous related research reports.^[24–26]^ The difference in the detection rate of anxiety and its severity may be related to factors such as the survey target population, the sampling method, and the specific time of the survey. Under the new the COVID-19 epidemic, the incidence of anxiety among college students is relatively high, indicating that the pressures such as the risk of illness and changes in teaching order under the background of the COVID-19 epidemic have indeed increased students’ anxiety and psychological problems.^[27–29]^ Countermeasures are needed to minimize the negative psychological impact of COVID-19 epidemic on college students. We have found that senior year, online game, relatives or friends are infected with COVID-19 are the independent risk factors of anxiety in college students after returning to school during the COVID-19 epidemic, and daily exercise is the independent protective factors of anxiety in college students after returning to school during the COVID-19 epidemic. School administrators and teachers should take early psychological interventions and care for college students based on these characteristics and influencing factors to reduce the occurrence of college students’ anxiety and improve students’ mental health.

Our findings have showed that senior students are more prone to anxiety. Senior students are faced with problems such as graduation thesis, employment, postgraduate entrance examination, and emotions, thereby they are under high psychological pressure. Moreover, the autumn semester of each year is the peak period for college graduates to seek employment. During the COVID-19 epidemic, in order to prevent and control the cluster infection, various arrangements have been postponed again and again, and many graduates have experienced the delay in completing the graduation defenses and employment.^[30–32]^ So graduates under the epidemic are at higher risk of developing anxiety. Therefore, school administrators and teachers should pay attention to the psychological comfort of senior students, make arrangements and notices for senior students’ graduation matters, and strengthen the connection of senior students’ career arrangements to reduce students’ anxiety level.

Our findings have found that that online gaming has a significant effect on the anxiety level of college students. The development of the internet is a double-edged sword. It can provide information and bring convenience to life, but it also has some negative effects.^[33,34]^ College students are not yet mature in physical and mental development, and lack the correct understanding and coping ability of the online world, and are easily addicted to it or affected by online information.^[35–37]^ Studies^[38,39]^ have shown that being addicted to the internet can induce anxiety in individuals. During the COVID-19 epidemic, college students have more free time and less academic pressure. They are easily addicted to the Internet, and receive information about the epidemic faster and easily. Excessive entertainment and miscellaneous online information may induce anxiety among students.^[40,41]^ It is necessary to control and reduce college students’ online gaming to reduce anxiety in college students.^[42]^ In addition, students with infected relatives and friends are at higher risk for anxiety and depression. New coronary pneumonia has a long incubation period and is relatively susceptible.^[43–45]^ When relatives and friends are infected, many people may fall into panic and fear. Therefore, the risk of anxiety among college students in this situation is significantly increased. Therefore, special attentions are needed to those students whose relatives or friends are infected with COVID-19.

We have found that students who exercise daily during school had lower anxiety and depression scores than students who do not exercise. Studies^[46,47]^ have shown that exercise can increase the secretion of dopamine, serotonin, and brain-derived neurokines in the brain to improve brain function for emotional regulation. Therefore, exercise can not only strengthen the body, but also relax the mind and have a positive impact on mental health. Previous studies^[48,49]^ have pointed out that healthy lifestyle habits such as regular work and rest and high-frequency physical exercise can also reduce students’ anxiety. Besides, the physical exercise can improve sleep quality. During exercise, the pituitary gland releases a large amount of endorphins with analgesic effect, which is conducive to the relief of anxiety. Exercise have a positive effect on the mental and intellectual state of college students, and help them rationally control various negative emotions caused by the epidemic.^[50]^ In addition, sports can also cultivate students’ mental toughness and improve their ability to cope with pressure, so as to cope with the COVID-19 epidemic with a more positive attitude.

Coping strategies for anxiety and depression of college students during the COVID-19 epidemic, college students are facing greater pressure than usual. Therefore, whether it is society, colleges or individual students, effective measures and nursing care should be taken in time to reduce anxiety. It is recommended that government administrators expand employment positions for graduates as needed, and employers launch online recruitment examinations and interviews to provide graduates with greater social support. Schools can offer career guidance courses for graduates, adopt online defense activities, and delay graduation if necessary to ensure students graduate normally.^[51]^ In addition, a psychological helpline or platform should be opened to provide students with professional help to relieve their psychological pressure and reduce the occurrence of psychological diseases.^[52]^ College students should arrange their life and study reasonably. Students should set daily learning goals, avoid indulging in online games, and insist on daily exercise to maintain physical and mental health. In addition, when they fall into fear, fear, depression, etc. and cannot self-regulate, college students should seek professional psychological assistance in a timely manner, such as contacting school psychologists or social psychological health consulting agencies, to avoid the negative impact of the continuous development of adverse psychology.

This study has certain limitations must be considered. Firstly, this study is based on a cross-sectional survey on the Internet in a short period of time. Due to the limitations of the research conditions, the research populations were not tracked longitudinally, so it was very difficult to understand the dynamic changes of anxiety among college students during the 2 years long COVID-19 epidemic. Secondly, the number of graduates participating in the survey was relatively small, the research area and population were limited, and the representativeness of the research results was limited. Therefore, larger sample survey in various areas are needed to further analyze the anxiety level and management strategies of college students in the context of the COVID-19 epidemic.

## 5. Conclusions

In conclusion, the results of this study show that during the COVID-19 epidemic, college students have a high incidence of anxiety. senior year, online game, relatives or friends are infected with COVID-19 are the independent risk factors of anxiety in college students, and daily exercise is the independent protect factors of anxiety in college students after returning to school during the COVID-19 epidemic. Universities, families, and society should take effective preventive measures in a timely manner. For graduates, as well as students with infected relatives and friends, psychological counseling should be provided when necessary, and students should be urged to reasonably arrange their study and entertainment life during COVID-19 epidemic. Besides, maintain adequate sleep, and conduct daily appropriate exercise may be beneficial to promote the physical and mental health of college students.

## Author contributions

SG designed research; TD, CZ, LJ, SG conducted research; TD, CZ, LJ analyzed data; TD, CZ wrote the first draft of manuscript; TD, SG had primary responsibility for final content. All authors read and approved the final manuscript.
